# Delayed Reperfusion—Coronary Artery Reperfusion Close to Complete Myocardial Necrosis Benefits Remote Myocardium and Is Enhanced by Exercise

**DOI:** 10.3389/fphys.2019.00157

**Published:** 2019-03-07

**Authors:** Eduardo C. A. Veiga, Ednei L. Antônio, Alexandra A. Santos, Brunno Lemes, Danilo S. Bocalini, Camila Picollo, Rosely F. Levy, Flavia L. Martins, Adriana Castello Costa Girardi, Andrey J. Serra, Paulo J. F. Tucci

**Affiliations:** ^1^Laboratory of Physiology and Cardiac Pathophysiology, Department of Medicine, Federal University of São Paulo, São Paulo, Brazil; ^2^Center of physical education and sports, Federal University of Espírito Santo, Vitória, Brazil; ^3^Department of Physiology, Federal University of Paraíba, Paraíba, Brazil; ^4^Laboratory of Genetics and Molecular Cardiology, Heart Institute (InCor), University of São Paulo Medical School, São Paulo, Brazil

**Keywords:** myocardial infarction, delayed reperfusion, late reperfusion, exercise, ventricular performance, inotropism, molecular biology

## Abstract

The present study aimed to analyze the effects of reperfusion of a distant coronary artery on cardiac function, the ultrastructure, and the molecular environment of the remote myocardium immediately after the completion of myocardial regional necrosis: delayed reperfusion (DR). Additionally, the effects of prior exercise on the outcomes of DR were investigated. Female rats with permanent occlusion or delayed reperfusion were randomly assigned to an exercise (swimming, 1 h/day, 5 days/week for 8 weeks) or sedentary protocol. Thus, the study included the following four groups: sedentary permanent occlusion, exercise permanent occlusion, sedentary delayed reperfusion, and exercise delayed reperfusion. The descending coronary artery was occluded for 1 h. Reperfusion was confirmed by contrast echocardiography, and the rats were observed for 4 weeks. Permanent occlusion and DR caused similar myocardial infarction sizes among the four groups. Interestingly, exercise significantly decreased the mortality rate. Delayed reperfusion resulted in significant benefits, including enhanced hemodynamics and papillary muscle contraction, as well as reduced apoptosis and collagen content. Protein calcium kinetics did not change. Meanwhile, developed tension and the Frank–Starling mechanism were enhanced, suggesting that calcium sensitivity was intensified in myofilaments. Remarkable remote myocardial benefits occurred after distant DR, and prior exercise intensified cardiac recovery. Our findings provide valuable information about DR. Our data might explain the better clinical outcomes in recent studies showing that late reperfusion could improve heart failure in patients with myocardial infarction. In conclusion, DR has remote myocardial benefits, including inotropism enhancement, pulmonary congestion reduction, and collagen and apoptosis attenuation, which are enhanced by prior exercise.

## Introduction

Myocardial infarction (MI) is a common cause of morbidity and mortality (Li et al., 2013), and many studies have evaluated the pathophysiological events occurring after MI. Coronary intervention during the acute MI phase is considered important for mortality prevention, myocardial remodeling, and reduction in the incidence and severity of congestive heart failure (CHF). Early reperfusion has been shown to reduce areas of necrosis (Sadanandan and Hochman, 2000; Vetterlein et al., 2003; Armstrong et al., 2013; Jones et al., 2015). Earlier implementation of coronary reperfusion is associated with greater benefits for the heart (Hochman et al., 2006; Wang et al., 2014). It is advisable to perform coronary reperfusion within 6 h following coronary occlusion for the rescue of ischemic myocardial injury (Sadanandan and Hochman, 2000). Notwithstanding, there is no consensus about coronary reperfusion performed after 6 h. The recent European guidelines (Ibanez et al., 2018) consider reperfusion therapy as class 1A in all patients with a duration of <12 h and persistent ST-segment elevation with symptoms of ischemia. Nepper-Christensen et al. (2018) reported that substantial myocardial salvage could be achieved beyond 12 h of symptoms in patients with MI and signs of ongoing ischemia. Abbate et al. (2008), in a necropsy study, described lower ischemia and apoptosis perinecrosis in infarcted patients with an open coronary artery than in those with an occluded artery. Gierlotka et al. (2011) analyzed patients who underwent coronary angioplasty 12–24 h following the start of symptoms and described an improvement in mortality when reperfusion was performed 12 h after MI, in contrast to a conservative approach.

Medical practice after early-onset coronary reperfusion is surpassed is not yet well-established. There is a need to determine the most appropriate cardiac pathophysiologic sequence following coronary occlusion and subsequent reperfusion at different times after coronary occlusion. These conditions emphasize the value of transactional research to better understand cardiac evolution in this scenario.

Most of experimental research in myocardial reperfusion focus injury reperfusion as the main aim. It is numerous the number of papers determined to study early reperfusion. To our knowledge, only one experimental report assessed the cardiac effects of late reperfusion. Nakagawa et al. (2008) performed myocardial reperfusion in rats 24 h after coronary occlusion and noted morphological and functional benefits: myocardial scar was thicker, wall tension reduced, and dP/dt increased. Additionally, it appears that no study has focused on the remote myocardial effects of regional coronary reperfusion immediately after complete myocardial necrosis. Moreover, no previous study has assessed the effects of prior exercise on remnant myocardial tissue. The purpose of the present study was to analyze the effects of reperfusion on cardiac function, ultrastructure and molecular environment of the remote myocardium immediately after the completion of myocardial regional necrosis in rats. Since this set is so different from usual study of late reperfusion, we called this set as delayed reperfusion (DR). As no previous studies have assessed the effects of prior exercise on remnant myocardial tissue in delayed reperfusion, we also sought to evaluate the influence of prior exercise in the outcomes of DR.

To define the duration of ischemia in our protocol, we considered the results of the studies by Hedstrom et al. (2009) and dos Santos et al. (2009) showing that in rats, total necrosis due to coronary occlusion occurs in <1 h. In the rats, we conducted coronary reperfusion immediately after the assumed risk area accomplished total necrosis, that is to say, 1 h after coronary occlusion.

## Methods

### Animals

Female rats (Rattus norvegicus var. albinus, 12 weeks old, weighing 180–220 g) were randomly assigned to swimming (1 h/day, 5 days/week, 8 weeks) or sedentary protocols and subjected to permanent occlusion or DR, generating four experimental groups: sedentary permanent occlusion (SPO), exercised permanent occlusion (EPO), sedentary delayed reperfusion (SDR), and exercised delayet reperfusion (EDR). This study was conducted in accordance with the Guide for the Care and Use of Laboratory Animals published by the National Institutes of Health (publication No. 85-23, revised 1985), the policies and regulations of the United Kingdom described in the Journal of Physiology (Grundy, 2015), and the Ethical Principles of the Brazilian College of Animal Experimentation (COBEA). The protocol was approved by the Research Ethics Committee of the Federal University of São Paulo (UNIFESP), Brazil (CEP 0341/08).

### Exercise Training Protocol

Exercise training was performed in a swimming pool (diameter, 132 cm; depth, 80 cm) filled with tap water warmed to 32–34°C by a feedback-controlled electric heating coil. The water was maintained in continuous turbulence to provide uninterrupted exercise. To allow adaptation, swimming was limited to 10 min on the first day and increased by 10 min each day. Rats were subjected to 60 min/day of swimming, 5 days/week for 8 weeks, as described by Bocalini et al. (2010). In each exercise session, 8–10 rats were placed together in the swimming pool. During the exercise period, an age- matched sedentary control group was exposed to similar room noise and handling but remained in their cages during rest.

### Surgery for Induction of MI and Ischemia/Reperfusion

After the first 8 weeks of exercise or physical inactivity, the animals were anesthetized with ketamine (50 mg/kg) and xylazine (10 mg/kg). Thoracotomy was performed, and the left anterior descending coronary artery was isolated and occluded ~3 mm from the origin of the aorta, using a previously described method (Johns and Olson, 1954) for induction of MI. The ischemia/reperfusion protocol described by Himori and Matsuura (1989) was as follows. A prolene suture 6-0 was passed to isolate the coronary artery and its point prepared. Before occlusion, two cotton threads were introduced into the prolene thread loop. The ends of each cotton thread were transfixed to each side of the chest wall to provide traction. Thereafter, the coronary artery was ligated, and the chest was closed. After 1 h of ischemia, with the chest still closed, the outer ends of the cotton threads were pulled to promote the opening of the coronary artery, releasing the flow of blood and promoting reperfusion. Reperfusion procedures were confirmed by echocardiography (ECHO). Five minutes before reperfusion, ECHO was performed to characterize the hypokinesia or akinesia of the left ventricle (LV) antero- lateral wall-dependent coronary occlusion. Following removal of the cotton traction threads, rats underwent ECHO with contrast (Definity, 0.2 mL, Bristol-Myers Squibb, New York, NY, USA) injected intravenously to confirm that coronary flow was restored. Animals with no reperfusion (*n* = 47/156, 30%) were excluded from the study. During 24 h following coronary occlusion 48/109 (44%) rats have died. Animals with MI smaller than 37% of ventricular endocardial perimeter (20/61; 37%) were only considered for analyses of mortality and were not used for functional, histomorphometric and molecular evaluations. Following these procedures, animals remained sedentary for 4 weeks until the final analysis, utilizing 12 rats in SPO; 12 rats in EPO: 8 rats in SDR and 9 animals in EDR.

### Echocardiographic Measurements

ECHO analysis was applied 4 weeks after the MI procedure. Rats were anesthetized, and measurements were taken using a 12-MHz transducer connected to an HP Sonos-5500 echocardiograph (Hewlett-Packard, Philips Medical System, Los Angeles, CA, USA). MI size was evaluated from transversal two-dimensional views of the LV on the basal, mid-transversal, and apical planes. In the diastolic phase, measurements of the LV endocardial perimeter (EP) and the infarcted segment length (IS) were determined for each view. MI size for each segment (MIS) was expressed as a percentage of the LV perimeter and calculated as follows:

$$\begin{array}{l} \text{MIS}\left(\text{\%}\right)=\text{IS}/\text{EP}\times 100. \end{array}$$

As in our previous studies (Helber et al., 2009; Santos et al., 2009), only rats with large infarctions (>37% of LV) were evaluated. The diastolic (DA) and systolic (SA) transverse areas of the LV were measured using two-dimensional images of the three parasternal transverse planes. The final value was the arithmetic mean of the measurements of the three views. Systolic function was analyzed using the shortening fraction (SF) of the three transverse planes and calculated as follows:

$$\begin{array}{l} \text{SF}=\text{DA}-\text{SA}/\text{DA}\times 100 \end{array}$$

Pulsed Doppler analysis of the LV side of the mitral valve provided flow velocity data, which were utilized to determine diastolic function parameters (E waves, A waves, and the E/A ratio).

### Hemodynamic Analysis

The LV pressure of anesthetized rats was obtained by catheterization of the right carotid artery using a Millar catheter (Microtip®, 2F, Millar Instruments, Inc. Houston, TX, USA). Data were obtained using AcqKnowledge® 3.7.5 software (Biopac System Inc, Los Angeles, CA, USA) to compute the instantaneous LV systolic (LVSP) and diastolic (LVDP) pressures, heart rate, and positive (+dP/dt) and negative (–dP/dt) first time derivatives (mmHg/s).

### Isolated Papillary Muscle Mechanics

Immediately after measurement of hemodynamics, hearts were removed and placed in oxygenated Krebs solution (all mmol/L: NaCl, 132; KCl, 4.69; CaCl_2_, 1.5; MgSO_4_, 1.16; KH_2_PO_4_, 1.18; C_6_H_12_O_6_, 5.50; HEPES, 20; pH, 7.40 ± 0.02;28°). As described in our previous reports (Bocalini et al., 2010, 2014), the posterior papillary muscle was dissected carefully from the left ventricle, mounted between two spring clips, and placed vertically in a chamber containing Krebs solution oxygenated with 100% O_2_. The upper portions of the muscles were attached to an isometric transducer (model FT03E; Grass Instrument, Quincy, MA, USA) connected to a micrometer for adjustment of muscle length. Preparations were stimulated 12 times/min with 5 ms square-wave pulses. The preparations were then permitted to contract isotonically under light loading conditions (0.4g) during a 60-min equilibration period. Finally, papillary muscles were loaded to contract isometrically for 15 min and stretched to the apices of their length-tension curves (L_max_). The following parameters were measured during isometric contractions: peak developed tension (DT), resting tension (RT), maximum rate of tension development (+dT/dt), and tension decline (–dT/dt). The mechanical behavior of the papillary muscles was evaluated under basal conditions and at 92, 94, 96, 98, and 100% of L_max_, allowing Frank-Starling curves to be determined, as previously described (Bocalini et al., 2010). Thereafter, muscle length at L_max_ was measured, and the muscle between the two clips was blotted dry and weighed. Muscle cross-sectional area (CSA) was calculated from muscle weight and length, assuming cylindrical uniformity and a muscle density value of one.

### Biometric Analysis

Wet and dry weights of the right lung were obtained, and the lung water content (%H_2_O) was determined as follows:

$$\begin{array}{l} \text{\%}{\text{H}}_{2}\text{O}=\left(\text{wetweight}-\text{dryweight}/\text{wetweight}\right)\times 100 \end{array}$$

Myocardial hypertrophy was estimated by dividing the heart weight by the body weight and defining the cardiomyocytes nuclear volumes of each rat, as is described in histomorphometric analysis.

### Histomorphometric Analysis

Sections of paraffin embedded tissue (4 μm) from the remnant myocardium were stained with picrosirius red to determine collagen content using an Aperio ScanScope Console v.8.0.0.1058 (Aperio Technologies, Leica Byosistem Inc, Los Angeles, CA, USA) and a published algorithm (Samuel, 2009). Perivascular fibrosis, defined as areas of stained intramural coronary arteries, were disregarded.

Hematoxylin-eosin staining was used for defining nuclear volume. The average nuclear volume (NV) was determined randomly in 50–70 myocytes cut longitudinally for each animal, and calculated according to the following equation (Gerdes et al., 1994):

$$\begin{array}{l} \text{NV}=\pi \times \text{D }\times {\text{d}}^{2}/6 \end{array}$$

where D = longer nuclear diameter and d = shorter nuclear diameter.

### Terminal Deoxynucleotidyl Transferase dUTP Nick end Labeling (TUNEL)

The apoptosis rate in the remnant myocardium was determined using TUNEL assays (Roche Applied Science, South San Francisco, CA, USA), according to the manufacturer's instructions. Six micrographs were randomly selected from each animal, and the numbers of healthy and apoptotic cardiomyocytes were counted. The percentage of apoptotic TUNEL-stained cells was calculated as a ratio of the total cell number.

### Preparation of Heart Homogenates, SDS PAGE, and Immunoblotting

Rat hearts were minced with razor blades and homogenized in a Polytron PT 2100 homogenizer (Kinematica, AG, Switzerland) in ice-cold buffer (pH 7.4) containing 150 mM NaCl, 7.2 mM Na_2_HPO_4_, 2.8 mM NaH_2_PO_4_, 15 mM NaF, 50 mM Na_4_O_7_P_2_ × 10H_2_O, and Halt Protease Inhibitor Cocktail (Thermo Fisher Scientific, Rockford, IL, USA). The Lowry method was used for protein quantitation. Homogenates were diluted in Laemmli buffer, boiled for 5 min, and proteins (60 μg) were separated by electrophoresis on 10% polyacrylamide mini-gels before electro-transfer to polyvinylidene difluoride (PVDF) membranes. Membranes were blocked with 5% skimmed milk for 2 h at room temperature and probed with primary antibodies, including anti-sodium/calcium exchanger (NCX) (1:1,000; Merck, Darstadt, Germany), anti-SERCA2 (1:1,000; Merck), anti-phospholamban (PLB; total) (1:500; Abcam, Santa Cruz Biotechnology, CA, USA), antibodies specific for PLB phosphorylated on Ser16 (1:500,000; Abcam, Santa Cruz Biotechnology, CA, USA) and Thr17 (1:100,000; Abcam, Santa Cruz Biotechnology, CA, USA), anti-mouse ryanodine receptor (1:5,000; Abcam, Invitrogen, San Diego, CA, USA), anti-caspase3 (1:500; Sigma-Aldrich, Invitrogen, San Diego, CA, USA), and anti-GAPDH (1:10,000; Abcam, Cambridge, USA), diluted in TBS-T buffer [50 mM Tris–HCl, 154 mM NaCl (pH 7.5), 0.1% Tween 20] plus 2% skimmed milk. After a 12 h incubation at room temperature, membranes were washed in TBS-T three times for 10 min and then incubated for 2 h with HRP-conjugated secondary antibodies (Invitrogen, CA, USA) diluted 1:1,000 in TBS-T plus 2% skim milk (Vector Laboratories, Los Angeles, CA, USA). Bound antibodies were detected using enhanced chemiluminescence reagent for 1 min. Bands were visualized and digitized using the ImageScanner LAS4000 mini (GE HealthCare, Little Chalfont, UK) and quantified using ImageJ software (Bethesda, MD, USA).

### Statistical Analyses

Data are presented as mean ± standard error of the mean (SEM). The Kolmogorov–Smirnov test was used to estimate Gaussian distributions. Levine's test was used to characterize the homogeneity of variances. Kaplan-Meier curves were used to define mortality. Two-way ANOVA and Bonferroni tests were applied to parametric data. Kruskal-Wallis analysis with associated Dunn's test was performed for non-parametric data. Analyses were performed using SPSS 12.0 (Systat Software Inc., Richmond, CA, USA) and GraphPad Prism 5.0 (GraphPad Software Inc., San Diego, CA, USA), and *p* < 0.05 was considered significant.

## Results

The reperfusion procedure was unsuccessful in 47 (30%) of the 156 animals subjected to temporary coronary occlusion.

### Mortality

**Figure 1:**
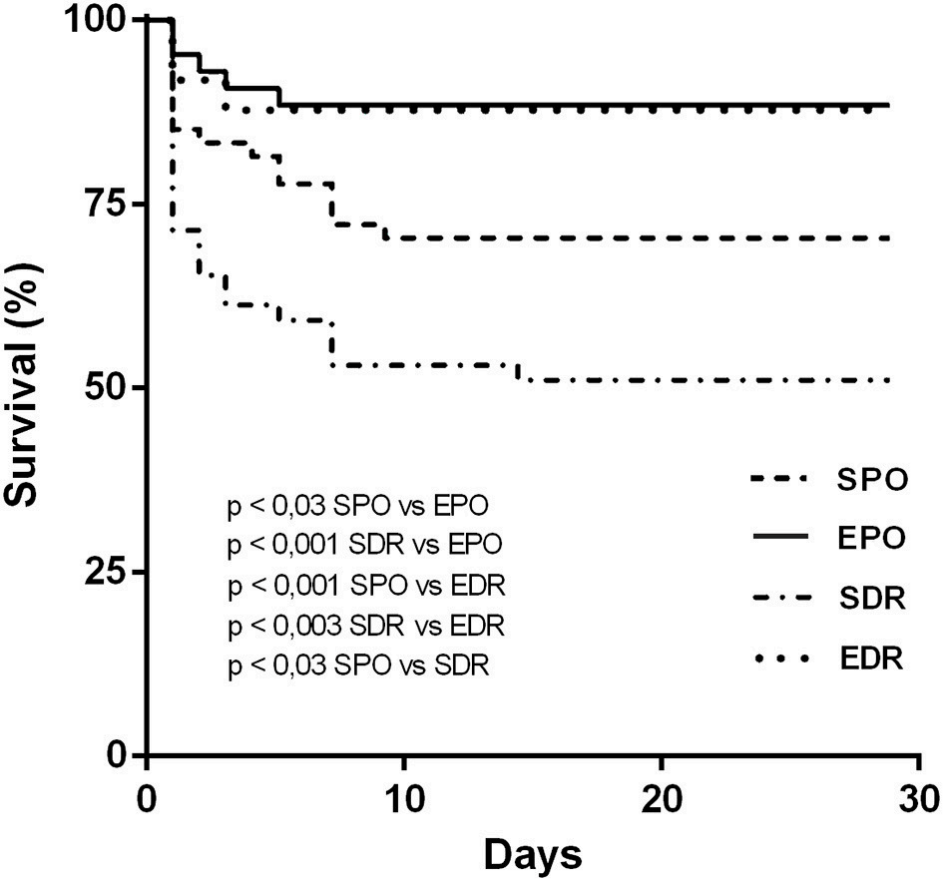
Kaplan–Meier curves illustrating the probability of survival vs. time in animals from sedentary permanent occlusion (SPO), exercised permanent occlusion (EPO), sedentary delayed reperfusion (SDR), and exercised delayed reperfusion (EDR) groups. Differences were evaluated using the log-rank (Mantel-Cox) test.

Kaplan-Meier analysis demonstrated that the survival of rats exercised prior to coronary occlusion was prolonged compared to sedentary rats (Figure 1). Expressive mortality occurred during 24 h following coronary occlusion. As reported in the literature (Neri et al., 2017), myocardial reperfusion frequently promotes arrhythmias, that can explain lower survival in this group. After 10 days of MI, survival curves were parallels. Differences in early mortality resulted in mortality differences between groups. Four weeks after coronary occlusion, the survival rates of rats in the EPO (11/12; 92%) and EDR (19/22; 86%) groups were higher than those of rats in the SPO (32/50; 64%) and SDR (14/28; 50%) groups. In addition, SPO survival was higher than SDR. However, survival was not significantly different between the two groups of exercised rats: EPO and EDR.

### MI Size, Pulmonary Congestion, and Biometrics

MI size, pulmonary water content, and biometrics are detailed in Table 1. There were no statistically significant differences in infarct size among the four groups. Lung water content, a measure of pulmonary congestion, was significantly lower in the EDR group compared with the sedentary groups (SPO and SDR), whereas the EPO group did not differ from the other groups. To evaluate myocardial hypertrophy, the average myocardial mass and nuclear volume from each group were assessed. Myocardial mass did not differ significantly among the groups, but nuclear volume was significantly lower in the exercised groups (EPO, EDR) than in sedentary animals (SPO, SDR).

**Table 1:** Cardiac parameters (mean ± SEM) of exercised and sedentary rats subjected to MI with or without LR.

	**SPO (*n* = 12)**	**EPO (*n* = 12)**	**SDR (*n* = 8)**	**EDR (*n* = 9)**
MI size (%)	44 ± 1	47 ± 2	46 ± 1	49 ± 2
DA (mm^2^)	0.59 ± 0.03	0.61 ± 0.02	0.63 ± 0.06	0.54 ± 0.02
SA (mm^2^)	0.54 ± 0.03	0.43 ± 0.03	0.42 ± 0.06	0.40 ± 0.03
SF (%)	30 ± 0.02	30 ± 0.03	35 ± 0.04	31 ± 0.02
E/A ratio	5 ± 0.5	5.8 ± 0.24	5.2 ± 0.6	4.4 ± 0.6
LVSP (mmHg)	98 ± 4	112 ± 5^*^	119 ± 5^*^	113 ± 5^*^
LVDP (mmHg)	22 ± 2	24 ± 1	20 ± 1	15 ± 1^#^
+dP/dt (mmHg/s)	5,052 ± 368	5,453 ± 198	6,719 ± 594^*^^#^	6,856 ± 121^*^^#^
–dP/dt (mmHg/s)	3,165 ± 243	3,833 ± 156^*^	4,023 ± 190^*^	4,290 ± 488^*^
LV weight (mg)	0.69 ± 0.01	0.58 ± 0.03	0.65 ± 0.04	0.63 ± 0.01
RV weight (mg)	0.20 ± 0.02	0.21 ± 0.01	0.23 ± 0.02	0.19 ± 0.01
%H_2_O	80 ± 0.4	79 ± 0.1	80 ± 0.5	78 ± 0.2^*^^&^
NV (μm^3^)	309 ± 15	203 ± 17^*^	257 ± 24	182 ± 16^*^^&^
Collagen (%)	6.29 ± 1.08	6.31 ± 1.29	2.27 ± 0.44^*^^#^	2.41 ± 0.38^*^^#^

*SPO, sedentary permanent occlusion; EPO, exercised permanent occlusion; SDR, sedentary delayed reperfusion; EDR, exercised delayed reperfusion; MI size, infarct size; DA, diastolic left ventricular area; SA, systolic left ventricular area; LV weight, left ventricle weight; RV weight, right ventricle weight; LVSP, left ventricular systolic pressure; LVDP, left ventricular diastolic pressure; +dP/dt, positive first time derivate; –dP/dt, negative first time derivate; %H_2_O, lung water content; NV, nuclear volume; Collagen, collagen content; SF, shortening fraction E/A ratio. Differences were evaluated by two-way ANOVA followed by post-hoc Bonferroni test*.

* *p < 0.05 vs. SPO*;

# *p < 0.05 vs. EPO*;

& *p < 0.05 vs. SDR*.

### ECHO and Hemodynamics

No differences in diastolic and systolic area, shortening fraction, or E/A ratio were observed between the groups by ECHO (Table 1).

The LVSP of the SPO group was lower than those of the other groups (Table 1), whereas the LVDP of the EDR group was lower than that of the EPO group. There were no other differences in LVDP values among the groups. Notably, DR enabled higher values of +dP/dt than those of animals subjected to permanent occlusion.

### Mechanical Response of Papillary Muscles

**Figure 2:**
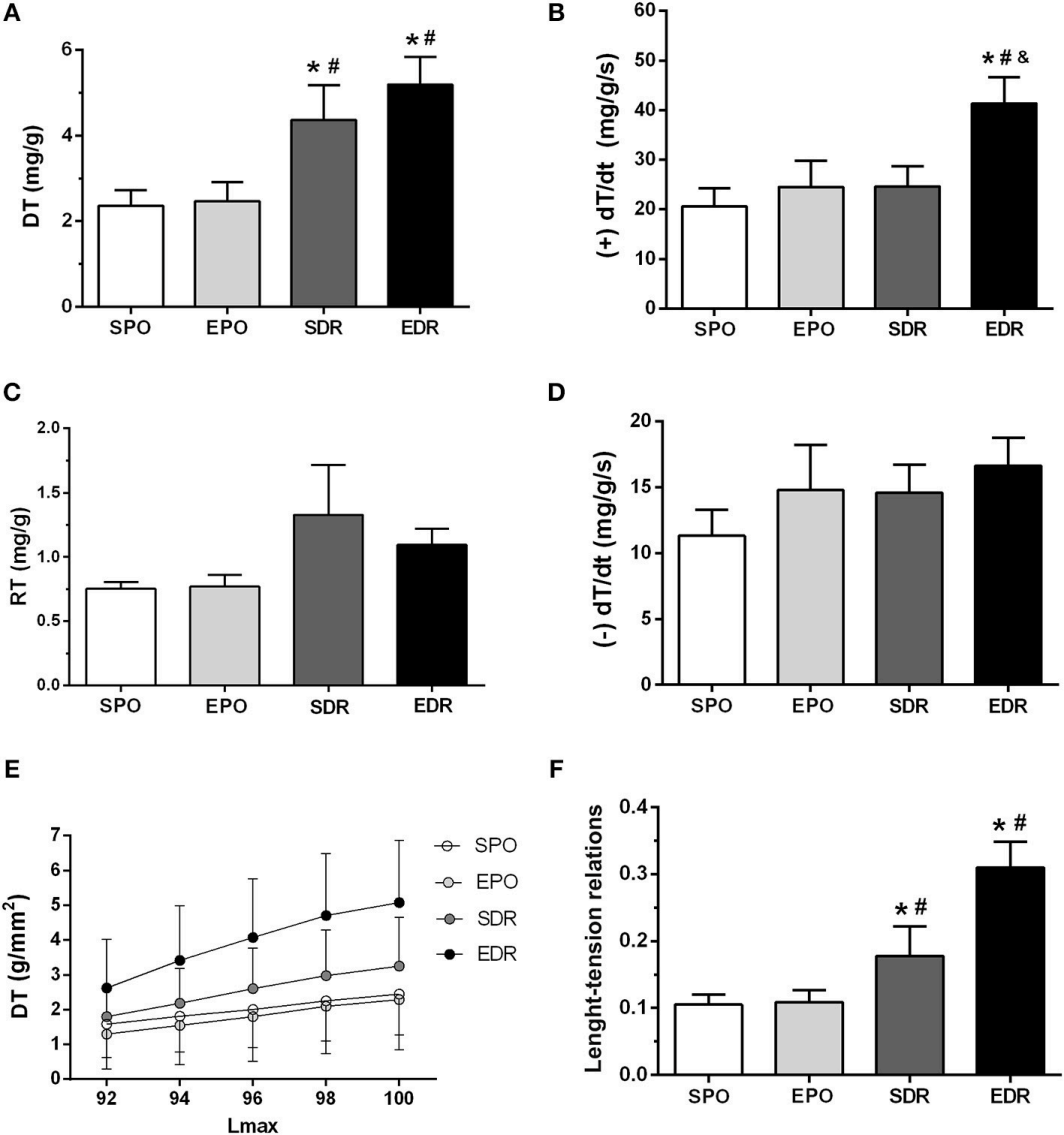
Papillary muscle parameters. **(A)** DT, developed tension. **(B)** +dT/dt, maximum positive derivative. **(C)** RT, resting tension. **(D)** –dT/dt, maximum negative derivative. **(E)** Plots of DT (g/mm^2^ × %Lmax). **(F)** Length-active tension relations slopes. Differences were evaluated by two-way ANOVA followed by *post-hoc* Bonferroni test. *p < 0.05 vs. SPO; ^#^*p* < 0.05 vs. EPO; ^&^*p* < 0.05 vs. SDR.

Posterior papillary muscles were isolated to examine their mechanical behavior in response to exercise and permanent or temporary coronary occlusion. There were higher DT in both reperfused group of rats than in rats subjected to permanent occlusion (Figure 2). Values of +dT/dt were elevated in EDR rats compared to those in the other groups, whereas, in respect to –dT/dt, there was no significant difference among groups. There were also no significant differences among RT of the four groups. In addition, the length- active tension ratios, which are indicative of the Frank-Starling mechanism, were steeper in the DR groups than the permanent occlusion groups. Altogether, the mechanical behavior exhibited by posterior papillary muscles suggests that muscle contraction is enhanced in DR animals.

### Collagen and Apoptosis in the Remaining Myocardium

**Figure 3:**
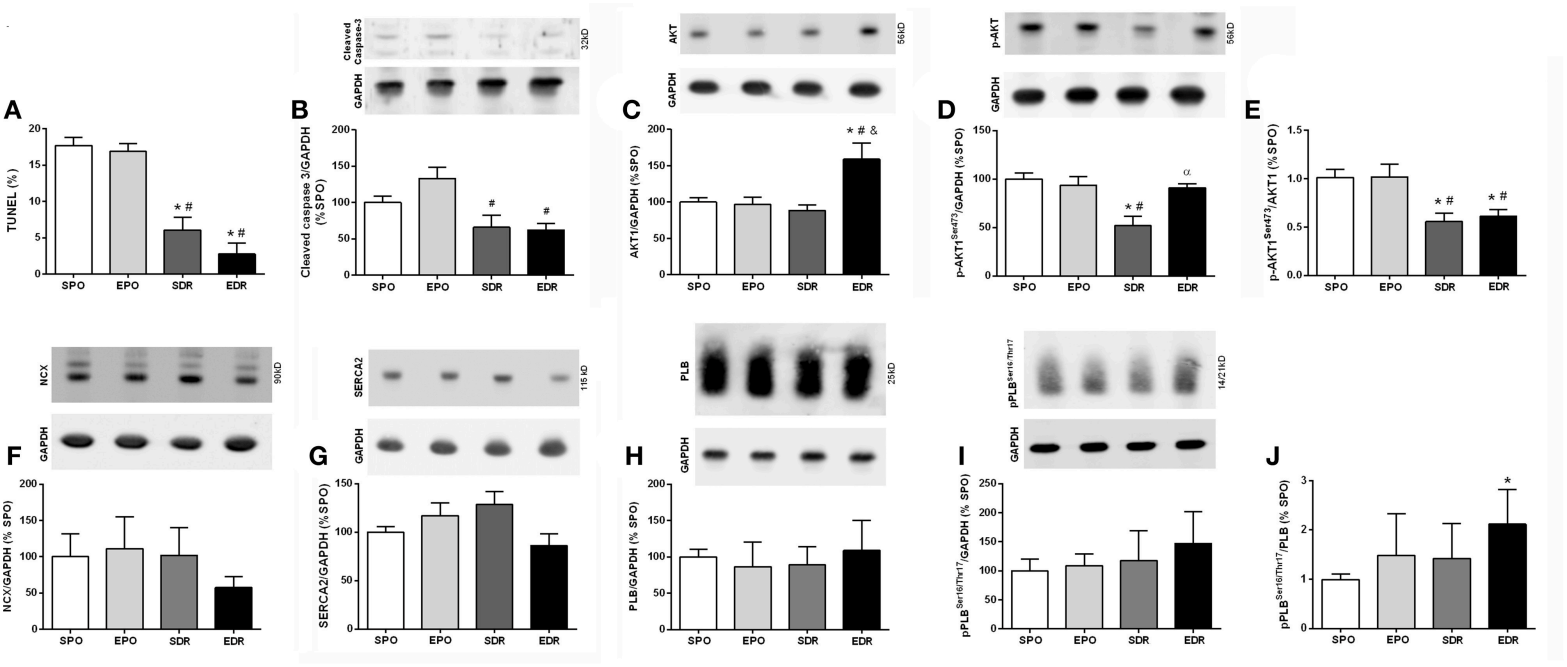
Apoptosis and calcium kinetics in sedentary permanent occlusion (SPO), exercised permanent occlusion (EPO), sedentary delayed reperfusion (SDR), and exercised delayed reperfusion (EDR) groups. **(A)** Percentage of apoptotic cells by TUNEL analysis. **(B)** Cleaved caspase-3. **(C)** AKT1. **(D)** Phosphorylated AKT1 (pAKT1). **(E)** Ratio of pAKT1/AKT1. **(F)** Sodium calcium exchanger (NCX). **(G)** SERCA2. **(H)** Phospholamban (PLB). **(I)** Phosphorylated PLB (pPLB) at serine (ser) 16 (PLB ser16) and threonine (thr) 17 (PLB thr 17). **(J)** Ratio of pPLB/PLB. Differences were evaluated by two-way ANOVA followed by *post-hoc* Bonferroni test. *p < 0.05 vs. SPO; ^#^*p* < 0.05 vs. EPO; ^&^*p* < 0.05 vs. SDR; ^α^*p* < 0.05 vs. EDR.

Collagen levels (Table 1) were significantly lower in the reperfused rats than those of the SPO and EPO groups. Moreover, apoptosis was significantly reduced in the EDR and SDR groups compared to the permanent occlusion groups, indicated by reduction of TUNEL positive cells (Figure 3A) and decreased cleaved caspase 3 (Figure 3B).

### Myocardium Protein Levels

There were no significant differences among groups in respect to calcium kinetics proteins (Figure 3): sodium/calcium exchanger (Panel C), SERCA 2 (Panel D), total PLB (Panel E), and p-PLB (Panel F).

## Discussion

Myocardial necrosis following coronary occlusion poses a serious risk to human lifes and motivates reperfusion. When not possible early reperfunsion, late reperfusion should be considered. Late reperfusion has been shown, in some recent studies, to benefit ischemic symptomatic patients reperfused more than 12 h after the onset of myocardial infarction (Ibanez et al., 2018; Nepper-Christensen et al., 2018; Yang et al., 2018). In infarcted patients without symptoms of residual myocardial ischemia there is no clear evidence of late reperfusion benefits. We analyzed the model of delayed reperfusion in sedentary and exercised rats for examination of mortality, cardiac remodeling, function, histology and molecular composition of remnant myocardium.

Our data showing comparable myocardium infarction sizes in permanent and temporarily coronary occluded rats indicate that 1 h of coronary occlusion successfully promotes total necrosis of risk areas in the rat heart. These results are in accordance with previous reports (Hedstrom et al., 2009) and support our previous findings (dos Santos et al., 2009).

Previous papers of Nakagawa et al. (2008) and Takemura et al. (2009) reported benefits in late reperfused compared to those with permanent occlusion; there is no notice about mortality. Our results related to mortality are comparable to several human studies that indicated no benefits on survival for patients who received coronary late reperfusion (Moreno et al., 1994; Horie et al., 1998; Yousef and Marber, 2000). Nevertheless, our Kaplan- Meier analysis demonstrated that previous swimming prolonged the survival of rats following temporary and permanent occlusion. Exercise prior to infarction has been reported to decrease mortality of infarcted rodents (Dayan et al., 2005; De Waard and Duncker, 2009).

In the current study, there were no statistically significant differences in cardiac mass of the right and left ventricles between groups, consistent with our previous findings (Veiga et al., 2011, 2013). However, nuclear volume, a more appropriate index of myocardial hypertrophy (Mill et al., 2011; Bajgelmen et al., 2015), indicated attenuation of hypertrophy in the exercised groups compared with the sedentary groups. To our knowledge, no previous studies have analyzed nuclear volume using a similar experimental protocol.

Although ECHO did not identify any difference among groups, there were indications of improved ventricular performance and remnant myocardial inotropism in reperfused animals. For example, LVSP, +dP/dt, and papillary muscle DT were improved after DR. Moreover, exercise intensified remote myocardial advantages following DR. In fact, LV end diastolic pressure, pulmonary water content, nuclear volume and the papillary length-active tension relationship slope were ameliorated after exercise in DR animals. Remarkably, exercise did not result in functional benefits in rats with permanent occlusion.

Although our calcium kinetics proteins data showed no relevant differences among groups, maximum DTs were higher in reperfused rats, and length-active tension relationships disclosed a Frank-Starling mechanism enhancement. Overall, these results suggest that calcium sensitivity may be improved in myofilaments.

Finally, our histologic analysis revealed meaningful benefits of DR in the remote myocardium. Indeed, reduced cleaved caspase-3 and TUNEL staining are in accord with lower levels of apoptosis in DR groups. These results are unprecedented for late myocardial reperfusion. Moreover, the clear reduction in collagen content in the remote myocardium of rats subjected to DR relative to those in non-reperfused animal's highlights additional benefits of DR. There is one unique previous work analyzing collagen content after late reperfusion (Nakagawa et al., 2008), reporting reduction of collagen in late reperfused rats.

### Conclusion

The main findings of this study were: (1) clear signs of remodeling with pathological hypertrophy, systolic and diastolic cardiac dysfunction in animals subjected to permanent occlusion; (2) these parameters were minimized after DR; (3) great remote myocardial benefits in delayed reperfused animals, including inotropism enhancement, pulmonary congestion reduction, and attenuation of collagen, apoptosis; (4) prolonged survival in rats exercised prior to coronary occlusion.

### Clinical Implications and Study Limitations

As in all translational studies, certain limitations to translate the finding to clinical use should be applied. Occlusion of coronary artery due to plaque rupture is an entirely different situation than artery ligation in animals. In the clinical scenario, drugs are used, background of cardiovascular risk factors and age may differentiate MI evolution. As no human cellular data after late reperfusion is available for comparison with our results, it is not possible to directly translate to medical practice our data obtained using rats. However, our unprecedented findings recommend that the clinical practice should consider the effectiveness of coronary reperfusion in humans, when the time to complete necrosis is borderline.

## Author Contributions

EV: conception and design of the study, acquisition of data, drafting the article and revising it critically for important intellectual content, and final approval of the version to be submitted. EA, DB, CP, AAS, FM, RL, and BL: acquisition of data, interpretation of data, and final approval of the version to be submitted. AG: introduction of a new technique, drafting the article and revising it critically for important intellectual content, and final approval of the version to be submitted. AJS: drafting the article and revising it critically for important intellectual content and final approval of the version to be submitted. PT: conception and design of the study, drafting the article and revising it critically for important intellectual content, and final approval of the version to be submitted.

### Conflict of Interest Statement

The authors declare that the research was conducted in the absence of any commercial or financial relationships that could be construed as a potential conflict of interest.
